# Mapping gene regulatory networks from single-cell omics data

**DOI:** 10.1093/bfgp/elx046

**Published:** 2018-01-12

**Authors:** Mark W E J Fiers, Liesbeth Minnoye, Sara Aibar, Carmen Bravo González-Blas, Zeynep Kalender Atak, Stein Aerts

**Affiliations:** 1 VIB Center for Brain & Disease Research, Laboratory of Computational Biology, Leuven, Belgium; 2 KU Leuven, Department of Human Genetics, Leuven, Belgium

**Keywords:** single-cell transcriptomics, single-cell epigenomics, gene regulatory networks

## Abstract

Single-cell techniques are advancing rapidly and are yielding unprecedented insight into cellular heterogeneity. Mapping the gene regulatory networks (GRNs) underlying cell states provides attractive opportunities to mechanistically understand this heterogeneity. In this review, we discuss recently emerging methods to map GRNs from single-cell transcriptomics data, tackling the challenge of increased noise levels and data sparsity compared with bulk data, alongside increasing data volumes. Next, we discuss how new techniques for single-cell epigenomics, such as single-cell ATAC-seq and single-cell DNA methylation profiling, can be used to decipher gene regulatory programmes. We finally look forward to the application of single-cell multi-omics and perturbation techniques that will likely play important roles for GRN inference in the future.

## Introduction

Gene regulatory networks (GRNs) define and maintain cell-type-specific transcriptional states, which in turn underlie cellular morphology and function. Each cell type or stable state is defined by a particular combination of active transcription factors (TFs) that interact with a set of *cis*-regulatory regions in the genome—in an interplay with chromatin structure—to produce a specific gene expression profile [1]. The combinations of active TFs and their target genes are usually represented as GRNs. Unravelling GRNs is one of the major challenges in the field of genome research. Once key regulators that drive and maintain the behaviour of a cell state are identified, they can ultimately be used to interfere with these regulatory programmes. Examples include reprogramming fibroblasts to induced pluripotent stem cells (iPS) by the combination of TFs proposed by Yamanaka *et al.* [2], many other reprogramming routes that steer a GRN from one state to another using specific combinations of TFs [3, 4] and recent attempts in cancer therapy, in which cancer cells are pushed into a state that is vulnerable to a particular drug [5, 6].

The computational prediction of GRNs based on large-scale transcriptome and epigenome data is an extensively studied field [6–8]. However, bulk technologies, such as microarrays, RNA sequencing (RNA-seq), DHS-seq, ATAC-seq or the different methylation-seq methods, measure the average signal from all the cells in a tissue or sample, which is in many cases composed of diverse cell types. While in some cases it is possible to extract specific cell types from a tissue, for instance by FACS sorting, this requires prior knowledge of specific markers and does not allow to identify novel cell states. With single-cell technologies, we can now gather omics-data from individual cells, allowing unprecedented opportunities to study the heterogeneity in GRNs, and to unravel the stochastic (probabilistic) nature of gene expression and underlying regulatory programmes. For these reasons, the field of regulatory genomics is undergoing a strong shift towards single-cell methods.

**Figure 1.**
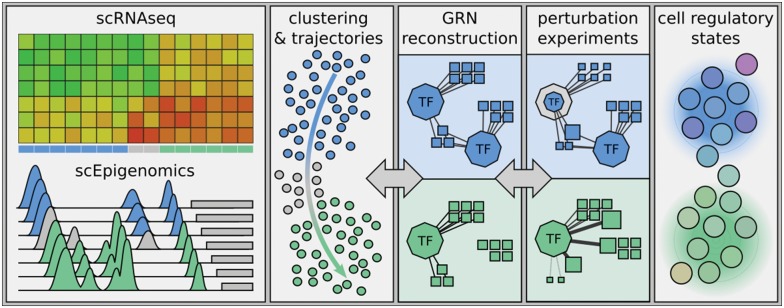
Single-cell GRNs. The goal of many single-cell studies is to understand which cell states are present in a heterogeneous sample; how these states differ from each other; how (and if) cells can switch from one state to another; and which states are relevant to the biological process under study. Cell states can be defined by GRNs, which can be inferred from scRNA-seq and scEpigenomics methods such as scATAC-seq and scMethyl-seq data. The two main classes of GRN inference methods are dynamic GRN methods that predict trajectories; and static GRN methods that can be used to predict cell states. Perturbation experiments can be used to confirm regulatory relationships.

In this review, we discuss how different single-cell omics techniques, together with computational methods, can be exploited to trace regulatory programmes across different layers: from the chromatin state in regulatory regions to GRNs (See Figure 1 for an overview). We will start with single-cell RNA-seq (scRNA-seq), currently the most broadly used and highest throughput technique, and explain how it can be used to detect sets of co-regulated genes and to infer potential master regulators. Moreover, we will describe how the latest developments exploit GRNs to cluster cells and decipher dynamic cell state transitions. Next, we discuss advances in single-cell epigenomic assays that provide a different approach to study gene regulation. We will cover in detail single-cell chromatin accessibility and single-cell methylation, as well as integrated approaches generating multiple read-outs per cell (multi-omics). The latter are particularly promising to ultimately lead to an integrated prediction of GRNs in the same cell, and may even bring the ultimate goal for a predictive model of gene expression within reach. Finally, we will cover single-cell perturbation assays that are being used to perturb GRNs (either at the level of TFs or enhancers) to study their influence on the transcriptome. These perturbation methods can be used to validate predictions, and potentially in the near future, they will become powerful tools for high-precision GRN inference. Overall, single-cell sequencing technologies—specifically scRNA-seq, single-cell ATAC-seq (scATAC-seq) and single-cell methylation profiling—already provide satisfactory data that enables network inference. They have successfully been used to infer regulatory associations in multiple studies, and even to study regulatory mechanisms [9]. Most other single-cell techniques were developed more recently and are still at the proof-of-concept stage. We expect that these methods, upon maturation, will become a disruptive tool in GRN inference, especially when combined with the development of new computational approaches. This will dramatically change how we study and understand GRNs, and ultimately cell states and state transitions.

## GRN inference from scRNA-seq data

scRNA-seq is the most frequently used single-cell sequencing technique today. After the first publication by Tang *et al.* [10] in 2009, many other methods have been introduced (reviewed by Svenson *et al.* [11]). Most methods follow a similar scheme, applying an adapted RNA-seq protocol to single cells that have been isolated and separated in droplets [12–15] or in microwells [16]. However, a transcriptome obtained from a single cell is currently not as sensitive or informative as its bulk counterpart: because of a combination of biological variation (e.g. stochasticity, bursts) and technical limitations, only a sample of the total mRNA population in a single cell will be captured, amplified and sequenced. The genes that remain undetected because of technical variation are referred to as dropouts [17, 18]. The level of dropouts is reflected by the median number of genes detected per cell (although this measure is confounding with the cell type), and usually forms a trade-off with the scale of the experiment (i.e. the number of cells sequenced). Larger numbers of cells sequenced will yield more statistical power to discriminate distinct cell states, and may compensate for some of the noise, but it will be hard to obtain conclusions for lowly expressed genes. This may be a particular problem when assessing TFs, which are typically lowly expressed [19].

After data processing, scRNA-seq data are represented as a counts table with the expression value for each gene in each cell. Most scRNA-seq analyses focus on the identification of cell (sub-) types or states within a population (e.g. in a tissue, or cancer), or along a dynamic process, such as differentiation (e.g. [20]), the cell cycle (e.g. [21]) or stimulus response (e.g. [22]). The computational methods used to solve these questions include clustering algorithms to group cells into distinct cell types or states (reviewed by Andrews *et al.* [23]) or trajectory inference methods to sort cells along a pseudo-time axis (reviewed by Canoodt *et al.* [24]).

**Figure 2.**
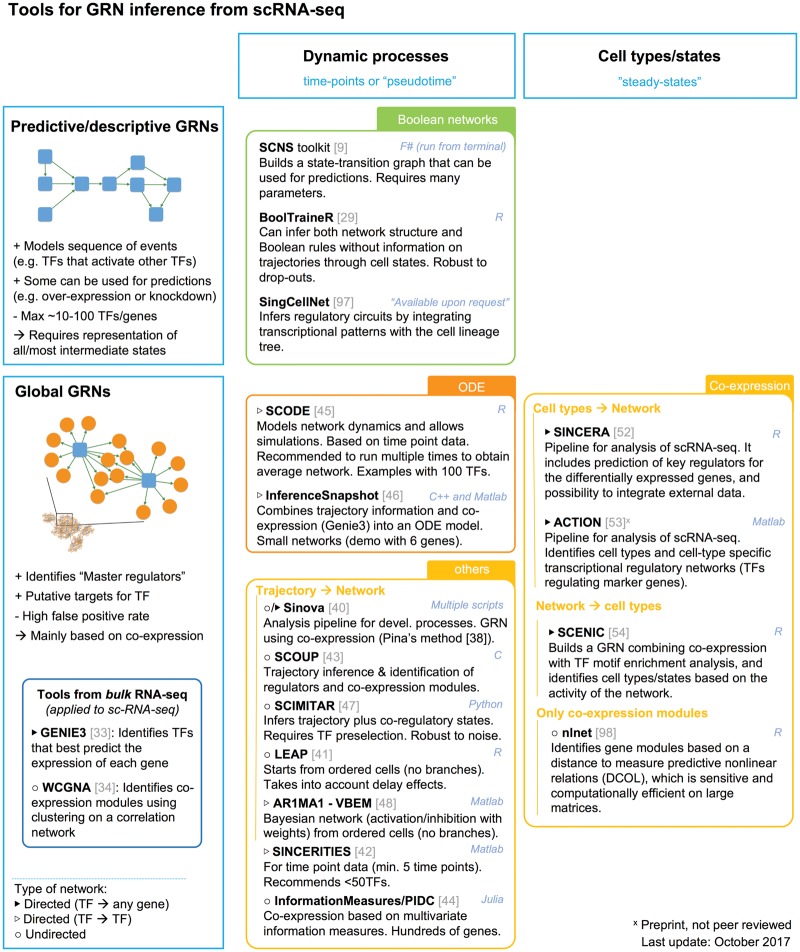
Tools for GRN inference from scRNA-seq data.

GRN inference from transcriptomics data typically relies on the assumption that regulatory information can be extracted from the expression patterns. For example, those genes with similar behaviours are regulated by a common mechanism, such as a specific TF. In this way, the aim of network inference can be (a) to model the sequence of TF activation events that lead from one state to another, (b) to identify potential targets for TFs or (c) to identify specific (combinations of) master regulators for a cell state. Many of the single-cell GRN inference methods (see Figure 2 for a summary) are based on the same principles as tools developed for bulk data (bulk methods are reviewed in [7, 25–27]).

One class of GRN inference methods focuses on deciphering the logical combinations of TFs required to transit from one state to another in a dynamic process. This is typically achieved through Boolean network models, like in Single-Cell Network Synthesis (SCNS) toolkit [28] and BoolTraineR [29]. Boolean networks are built by classifying each cell into a state (based on the TF expression) and connecting cells that have a limited number of differences. The resulting state-graph provides insight into key TFs involved in state changes, and can be used to predict the effect of over-expression or knock-down of a TF. However, it does not provide information about target genes. In addition, computational demands increase rapidly with network size; hence, these tools can model only a small number of genes (<100). For this reason, these approaches are normally applied to dynamic processes after a trajectory inference step and selecting a subset of relevant TFs. Another drawback of Boolean networks is the conversion of expression levels to a binary state (active/not active, based on a threshold), which makes them unable to reliably model dose response relationships [30], and they are sensitive to dropouts. Examples of applications of these methods include modelling the reprogramming of iPS [31] and the work of and Moignard *et al.* [9], where the authors modelled the regulatory network of blood development using a branched trajectory with diffusion maps and SCNS toolkit.

An alternative approach to regulatory network inference consists of linking TFs to candidate target genes with the ultimate goal of identifying ‘master regulators’ driving a specific cell state. A major family of methods in this category is based on co-expression analysis (reviewed in [32]), and has been broadly used on bulk gene expression data (e.g. GENIE3 [33] and WGCNA [34], see [27, 35] for benchmark comparisons). Recent studies have successfully applied similar approaches to single-cell data, for example, by Patel *et al.* [36] to identify gene signatures and TFs that correlate with the stemness gradient in glioblastoma; by Gaublomme *et al.* [37] to identify pathogenicity regulators that co-vary with pro-inflammatory genes; and by Pina *et al.* [38] to reconstruct a GRN of haematopoiesis based on pairwise associations. Elements that need to be taken into account when using these approaches include the assumption that variation in the expression level of the regulator directly influences the expression of downstream targets, ignoring (for example) post-transcriptional regulation, or the fact that not all co-varying genes are necessarily direct targets. In addition, these methods are sensitive to normalization and batch-effects, which may introduce artificial co-variation [39].

A specific subgroup of co-expression-based methods is geared specifically towards building GRN models from single-cell transcriptome data from dynamic processes. These methods combine an initial ordering of cells along a time axis (or a predicted trajectory) while modelling the expression dynamics between genes and regulators, using techniques such as (non-linear) correlation [40, 41], regression [42], covariance analysis [43], multivariant information [44], ordinary differential equation (ODE) models [45, 46] and others [44, 47, 48] (see Figure 2 for an overview). A special category within these is the ODE models, which reconstruct expression profiles over time to detect and merge similar profiles. The systems of ODEs allow inferring some causality, and are more realistic and detailed than alternative approaches. However, they require a large amount of input data to reliably estimate their parameters, and they are computationally intensive, so they can only be applied to a limited number of TFs and targets.

When studying a system without trajectory information, such as a tissue consisting of multiple distinct (static) cell types, a different approach needs to be taken. Studies characterizing the heterogeneity of complex tissues typically focus on identifying the cell types and the gene signatures or markers that characterize them (e.g. [49–51]), but further regulatory analysis is not common. Some recent tools that attempt to bridge this gap are SINCERA [52] and ACTION [53], two pipelines for the analysis of scRNA-seq data that include an extra step to search for key regulators of the cell-type-specific signatures. Finally, SCENIC [54]—developed in our laboratory—infers a GRN directly from the data, identifying direct regulatory interactions between TFs and targets by integrating co-expression with motif enrichment analysis. The activity of this GRN in each cell is then used to identify cell types/states, and key TFs that characterize each state with higher accuracy.

In conclusion, early results show that it is possible to reconstruct GRNs from single-cell transcriptomics data. Moreover, it can even be advantageous to use (predicted) regulatory relationships into the clustering of single-cell data, and it will ultimately be vital in understanding cellular heterogeneity.

## Single-cell epigenomics

Single-cell epigenomics provides a complementary description of transcriptional states, now represented as epigenomic landscapes. While the transcriptome is the result of transcription, post-transcriptional regulation and RNA degradation, the epigenome provides a perspective that is closer to the transcriptional process. An epigenome reveals which regulatory regions (e.g. enhancers and promoters) are operational in each state. Single-cell epigenomes are bound to increase the insight into the cellular (transcriptional) heterogeneity and are starting to provide valuable additions to scRNA-seq when mapping GRNs. Although multiple approaches for single-cell epigenomics exist, we will mainly discuss accessible chromatin profiling, specifically ATAC-seq, and DNA methylation because these have already been applied to multiple biological systems. Other epigenomic methods have also been adapted to single-cell levels, for instance, scDNase-seq [55] for open chromatin profiling, scDamID-seq [56] for mapping genome-nuclear lamina interactions and scChIP-seq [57] for TF binding or histone modifications. However, we will not discuss these, as they are still in the proof-of-principle phase or not widely adopted yet.

There are two experimental methods to perform scATAC-seq, either based on a microfluidic platform (Fluidigm C1) for physical isolation of single cells [58] or on combinatorial cellular indexing of sorted nuclei (sciATAC-seq) [59]. Both methods are derived from the original ATAC-seq protocol, using a hyperactive Tn5 transposase for simultaneously cutting and tagging accessible chromatin. These methods have been reviewed by Pott and Lieb [60]. There are three main differences between the microfluidics- and combinatorial indexing-based methods: (i) combinatorial indexing has a higher throughput of 500–1500 single cells per run, as compared with 96 cells with the microfluidics-based platform; (ii) but the number of reads per single cell reported by Cusanovich *et al.* [59] for combinatorial indexing (median of 2503 reads per cell) is considerably lower than those obtained via the microfluidic approach (73 000 reads per cell); and (iii) the microfluidics approach of Buenrostro *et al.* can use a commercially available Tn5 transposase, whereas combinatorial indexing requires a self-made Tn5 transposase tagged with different barcodes. Both methods have already been used to study gene regulation, e.g. Buenrostro *et al.* [58] showed that chromatin accessibility variance in K562 myeloid leukaemia cells was associated with specific TF-binding sites (including GATA1/2, JUN and STAT2). Cusanovich *et al.* illustrated that sciATAC-seq is not only able to uncover different combinations of TF activity between cell lines but also between cells within an apparently homogeneous population. For instance, by grouping coordinated combinations of accessible regions, they identify subtypes in the lymphoblastic cell line GM12878, of which the variability is driven by activity of the nuclear factor-kappa B pathway. Recently, scATAC-seq was also used to study the epigenomic landscape of human haematopoietic differentiation [58, 61]. These studies revealed regulatory heterogeneity within immunophenotypically sorted cellular populations that are governed by diverse regulatory programmes at individual *cis-*regulatory elements and by relatively simple TF motif dynamics. Interestingly, scATAC-seq was able to capture the distinct regulatory states and trajectories, leading to the finding of a continuous regulatory landscape that underlies human haematopoiesis [58].

It is important to note that single-cell chromatin accessibility data are even more sparse than scRNA-seq. As in a diploid genome there are (usually) two copies of a regulatory element, the read-out of chromatin accessibility is nearly binary, and has a large amount of dropouts. Therefore, these data require specialized analysis methods that generate accessibility measurements either across groups of cells [62] or across sets of genomic features (e.g. based on ChIP-seq data or TFs) [63]. For example, Cusanovich *et al.* [59] adapted a previously existing text clustering method called latent semantic indexing to unravel different cell types based on their accessibility profile patterns and to identify sets of co-accessible regions. An alternative approach is taken by scABC that applies an initial K-medoids clustering on read depth corrected data to extract ′landmarks′ or average profiles of the different cell clusters, which are used to reassign the cells to the clusters [62]. Similarly, Cicero [64] performs cell clustering on scATAC-seq trajectories (constructed with an adaptation of Monocle 2 [65]), determines co-accessible regions within each of these groups and finally infers chromatin hubs by taking into account the correlation between nearby regions. On the other hand, chromVAR uses predefined cistromes (defined as a set of *cis*-acting targets of a TF) or determines them *ab initio* based on motif enrichment to calculate average bias-corrected *Z*-scores [63]. Next to the computational challenges, there is still room for experimental improvements towards a sc-chromatin accessibility method that combines both high-throughput and high per-cell read coverage, ideally in a droplet microfluidic format to increase the scale. In any case, single-cell chromatin accessibility technologies are already great tools to study regulatory programmes in single cells.

A second epigenomics technique that is gaining traction is single-cell DNA methylation profiling. DNA methylation is one of the most studied regulatory mechanisms, especially in lineage commitment during development and diseases such as cancer [66, 67]. Increasing the resolution of DNA methylation measurements to the level of single cells allows the discovery of methylation marks that identify regulatory programmes underlying subpopulations or particular cell states. Several single-cell whole methylome technologies exist, namely, scRRBS-seq [68], scBS-seq [69], scWGBS-seq [70] and single-cell CpG island sequencing (scCGI-seq) [71]. scRRBS-seq is based on reduced representation bisulphite sequencing that enriches CpG dense sites in the genome [68]. Therefore, only a low number of reads are required to get a high coverage at CpG islands (CGIs), leading to a reduced cost per cell with high information of CpG-dense sites. However, this comes with the disadvantage that only 10% of all CpG sites in the genome (0.5-1.5 CpGs per single cell) is detected, and importantly, CGI are mostly found at gene promotors, whereas a large part of *cis*-acting regulatory elements (e.g. enhancers) are CpG-poor (reviewed by Jones *et al.* [72]). The same is true for scGCI-seq, which combines methylation-sensitive restriction enzyme digestion and multiple displacement amplification to selectively detect methylated CGIs, going up to 76% of all CGIs detected on average per cell with high consistency among cells [71]. Two other single-cell methylome methods (scBS-seq and scWGBS-seq) give a broader picture of the entire methylome by aiming to measure DNA methylation at all CpG sites (as opposed to only CGIs). scBS-seq [69] is best suited for deeply sequencing sc-methylomes with maximum coverage, whereas scWGBS [70] is optimized to profile many samples at low coverage. These two methods have been used to analyse DNA methylation heterogeneity at several classes of *cis-*elements, showing that regions with active enhancer marks have a high variance in DNA methylation between single cells [73], and regions with a quick loss of DNA methylation during cell state transitions contained lineage-specific enhancer elements and TF-binding sites [70]. These methods are already able to capture DNA methylation changes during a state transition by sampling at different time points. However, precise investigation of the dynamics of these epigenetic changes is cumbersome with sequence-based techniques. For this purpose, reporter-based assays are more suited. Stelzer *et al.* [74] established a reporter of genomic methylation (RGM), allowing real-time tracking of changes in DNA methylation at a specific locus, both *in vitro* and *in vivo*, with single-cell resolution. RGM was used to monitor the DNA methylation status of non-coding regulatory elements during different biological processes, such as cellular reprogramming, tracking the enhancers associated with Sox2 and miR290. They show that de-methylation of both these enhancers is a late event in the reprogramming process. As with other single-cell methods, there are different sc-methylation approaches yielding different results. Differences can be because of (different levels of) technical dropouts, variation in detection power (e.g. scRRBS is able to detect 10% of CpG sites [68], whereas scBS-seq can detect up to 48% [73]) and different analysis approaches. This makes it, without extensive benchmarking studies, difficult to determine which method performs best. One possible way to deal with technical dropouts is proposed by Angermueller *et al.* [75] by developing DeepCpG, a computational approach based on deep neural networks, to predict missing methylation states and link methylation states to motifs. They showed that regions with increased methylation variability associate significantly more strongly to gene expression, indicating functional relevance.

## Single-cell multi-omics

Transcriptome and epigenome provide different viewpoints of a GRN; therefore, obtaining both of them simultaneously from the same cell population, and ultimately from the same cell, would provide a comprehensive view of the cell’s regulatory states, and would allow studying the interplay between different regulatory layers. Litzenburger *et al.* [76] combined scATAC-seq and scRNA-seq data from the same cell population to study effects of variation in DNA accessibility at GATA-binding sites and identified co-varying expression of cell surface markers. However, computationally combining several omics data sets only provides indirect links. To obtain these measures from the same cell, several technologies are emerging [77, 78] (Table 2).

The first methods developed for integrated multi-omics allow parallel interrogation of the genome and transcriptome of the same cell (G&T-seq [79], DR-seq [80] and single-cell transcriptogenomics [81]). These methods typically focus on the transcriptomic consequences of chromosomal abnormalities such as copy number variation (CNV) or single-nucleotide variation. For example, Macaulay *et al.* [79], using G&T-seq, observed higher expression of genes on chromosome 11 in cells with trisomy of this chromosome, unambiguously linking chromosomal amplifications with effects on gene expression in a single cell.

Several other methods have been published to simultaneously measure several omics features from one cell (all of them including DNA methylation). scM&T-seq [82] measures DNA methylome and transcriptome via a similar protocol as G&T-seq for physically separating mRNA and DNA, but instead of performing WGS on the DNA fraction, it undergoes scBS-seq to profile the methylome. In contrast, scMT-seq [83] and scTrio-seq [84] use scRRBS-seq (for single-cell methylome profiling), after a mild ligation protocol that leaves the nuclear membrane intact so that the cytosolic fraction can be separated from the nucleus. In addition, scTrio-seq determines CNVs through computational analysis of the methylome data [84]. The last method, scNOMe-seq [85], achieves simultaneous measurement of DNA methylation and chromatin accessibility in single cells using a CpG methyltransferase that methylates CpGs in non-nucleosomal DNA followed by bisulphite sequencing [85]. Based on this, Clark *et al.* [86] developed scNMT-seq (a combination of scM&T-seq and scNOMe-seq), and show that, globally, single-cell methylation and open chromatin data are highly anti-correlated and thus contain similar information. Next to studying how different regulatory layers affect one another or looking at cellular heterogeneity between these different levels, most of these studies provide good examples on how single-cell multi-omics techniques improve our understanding of the role of epigenetic modifications of *cis-*elements in GRNs. For instance, Angermueller *et al.* [82] studied associations between changes in methylation and expression of individual genes. They detected 1493 associations, both positive and negative, highlighting the complexity of the methylome–transcriptome interaction. Negative correlations were predominantly found for non-CGI promotors, as expected from literature [72] and matching the findings from scMT-seq [83], but distal regulatory elements showed a more even distribution of positive and negative associations. scTrio-seq shows a correlation between CNV and mRNA expression, but no effect on DNA methylation. scNOMe-seq describes the relationship between DNA methylation and chromatin accessibility in single cells. By detecting footprints of TFs, scNOMe-seq predicts TF activity in individual cells, and how this is affected by DNA methylation, thus studying cell-to-cell variation at the regulatory level. These examples illustrate a great potential for single-cell multi-omics to build complex models of gene regulation. However, current multi-omics techniques still present a high cost per cell and have a limited throughput. This makes them currently rather suited for studying unique cells (e.g. embryos with chromosomal abnormalities) or rare (sorted) cell types. For large-scale studies, computational integration of independent layers of omics remains the only solution until droplet-based methods or methods based on combinatorial indexing can deliver single-cell multi-omics measurements. One example of a computational approach is shown by Welch *et al.* [87], using the MATCHER tool, which integrates multiple omics layers, e.g. scRNA-seq with scMT-seq. In summary, single-cell multi-omics promises to become a powerful method integrating multiple regulatory layers into GRNs.

## Single-cell regulatory perturbations

Perturbation experiments are an important tool to assess regulatory relationships between genes. For example, if the expression of a TF or the sequence of an enhancer is altered, the expression of the target genes is expected to be affected as well. Standard perturbation-based approaches are widely applied, but still expensive, time-consuming and difficult to parallelize. Several methods have been developed using pooled CRISPR/CAS9 genome editing to introduce a large amount of perturbations in a population of cells, the effects of which can subsequently be measured using single-cell transcriptomics. These methods include CRISP-seq [88], Perturb-seq [89, 90], CROP-seq [91] and Mosaic-seq [92].

One of the challenges of these methods was how to establish the link between cells and perturbations. Perturbations are induced by guide RNAs (gRNAs) that target genome locations to perturb TFs or enhancers. However, as gRNAs lack a poly-adenylated (poly-A) tail, they are not detected by scRNA-seq methods (as they are all polyA-based). To identify which perturbation is applied to each cell, CRISP-seq, Perturb-seq and Mosaic-seq use poly-A barcodes, which are then computationally linked to the gRNA. In contrast, CROP-seq includes the gRNA in a poly-A mRNA transcript, which is sequenced with the rest of the mRNA, simplifying the screening of larger libraries of gRNAs [93].

As all four methods are based on CRISPR/CAS9, in principle, any CRISPR-based perturbations could be used, including knockouts and transcriptional- or epigenetic-based repression or activation (e.g. CRISPRi, CRISPRa [94]). They can also be used to perturb promoters, enhancers or non-coding RNAs as well as protein-coding genes. Indeed, the related publications display a wide array of applications. CROP-seq was applied to characterize transcriptome changes after T-cell receptor pathway induction in Jurkat cells [91]. Using a gRNA library for 6 high-level regulators of the pathway and 23 TFs, the authors derived gene signatures for each of the perturbations in naive cells, and cells stimulated with anti-CD3/CD28 antibodies. Similarly, CRISP-seq and Perturb-seq were also applied in a knockout context focused on identifying key regulators of the immune response and their combinatorial effects. A companion paper of Perturb-seq uses CRISPRi to study the epistatic effects of three key regulators of independent pathways involved in unfolded protein response. CRISP-seq also shows that it is possible to study perturbations *in vivo*, by injecting transduced hematopoietic progenitor cells into mice. Finally, Mosaic-seq used dCas9 coupled with KRAB, a transcriptional repressor, to study the endogenous activity of enhancers and the individual contribution of the different constituents of large arrays of enhancers.

With scRNA-seq data being intrinsically noisy and containing numerous dropout events, the bioinformatics analysis of sc-perturbation assays is challenging. A barcode or gRNA can easily be missed, and hence, the measurements from an individual cell are not reliable. The CROP-seq authors opted for a simple approach: deriving the knockout signatures for each condition from the aggregate of cells targeted by the same gRNA. The CRISP-seq authors base their analysis on an unsupervised clustering method, assigning barcodes based on phenotypic similarity. Perturbation effects are calculated across groups of cells and controls. In Perturb-seq, a computational method is developed based on a linear model (Multi-Input-Multi-Output-Single-Cell-Analysis, MIMOSCA), which allows studying the impact of individual perturbations on gene expression and the marginal contributions of the genetic interactions. Finally, the authors of Mosaic-seq base their analysis on two key parameters that represent the enhancer activity in individual cells: the penetrance in the population and the contribution to expression in these cells.

In conclusion, these four approaches open exciting opportunities for the exploration of GRNs. They present interesting proof of principle applications, exploring the effect of a few dozen perturbations per experiment (typically targeting 10–30 genes, TFs or enhancers), and all expect that the number of elements screened will increase in future applications. One general challenge for these CRISPR methods involves the specificity and efficiency of gRNAs (reviewed in [94]). The aforementioned methods try to overcome this by increasing biological and technical replicates: designing multiple gRNAs per target (an average of three gRNAs per target) and transfecting multiple samples with the same gRNA. Another challenge is accurate detection of the gRNA barcode, which is limited by the sensitivity of the scRNA-seq approaches. Also, it is likely that future applications multiplex a number of gRNAs [95, 96] to test for specific combinations.

## Future perspectives

The single-cell field is under rapid development, both in terms of technological advances to acquire single-cell data, and computational solutions to identify biological novelties in the data. An important hurdle, currently being tackled at multiple fronts, is how to overcome the biological and technical noise in a single-cell data set. Given the experimental nature of methods reviewed here, and the speed with which developments take place, it will be interesting to see how future benchmarking studies, comparing different techniques, will eventually yield best practices.

In this review, we have particularly focused on methods that analyse GRNs and chromatin activity at the single-cell level, both as a means to identify stable cell states, and as an endpoint to unravel the genomic regulatory logic. Several studies have already predicted regulatory interactions from single-cell data, where the ability to study individual cells has been proven to outweigh the added noise. In the near future, a lot of work will be required to improve the reconstruction of global GRNs of large single-cell data sets. Specifically, focusing on methods to distinguish regulatory differences between (steady state) cellular (sub-) types, and to improve and upscale methods to predict the regulatory kinetics of dynamic processes. One particularly promising application is merging the prediction of gene regulatory dynamics with single-cell clustering and trajectory inference. The prediction of transcriptional regulation improves understanding of cellular heterogeneity, and *vice versa*. We expect that further improvements of single-cell epigenomics methods such as scChIP-seq [57] and scDamID-seq [56], and the upscaling of scATAC-seq and scMethyl-seq—for example towards droplet-based approaches—will provide an important push in the field. In addition, when multi-omics approaches get more traction and when they can be performed at lower cost and higher scale, they may provide the ultimate data towards understanding GRNs. Indeed, linking epigenome changes with transcriptome and proteome changes may lead to long-awaited single-cell systems biology solutions. A thorough understanding of GRNs will also help us to better understand cellular lineage, identity and variation. With this knowledge, we will be able to develop a completely new perspective on (for example) pathology, for instance by observing the first reaction of a cell to a pathogenic condition with unprecedented detail, but also by studying cellular population dynamics. In conclusion, single-cell technologies represent a disruptive technique in the prediction and understanding of GRNs, and of cellular biology in general.

**Key Points**

- The field of regulatory genomics is shifting towards single-cell resolution.
- GRNs can be reverse-engineered from scRNA-seq data.
- Single-cell GRNs are useful to identify stable cell states and cell state transitions.
- Single-cell epigenomics, single-cell perturbation assays and single-cell multi-omics provide exciting opportunities to unravel transcriptional programmes.
- Methods to map GRNs from scRNA-seq data are emerging quickly; methods to unravel regulatory programmes from single-cell epigenomics data are lagging behind.

## Funding 

This work is funded by The Research Foundation – Flanders (FWO; grants G.0640.13 and G.0791.14 to S. Aerts; G092916N to J.-C.M.), Special Research Fund (BOF) KU Leuven (grants PF/10/016 and OT/13/103 to S. Aerts), Foundation Against Cancer (2012-F2, 2016-070 and 2015-143 to S. Aerts) and ERC Consolidator Grant (724226_cis-CONTROL to S. Aerts). L. Minnoye is funded by a FWO-SB PhD fellowship and a Kom Op Tegen Kanker fellowship. S. Aibar is supported by a PDM Postdoctoral Fellowship from the KU Leuven. Z.K.A. is supported by a postdoctoral fellowship from Kom op Tegen Kanker. The funders had no role in study design, data collection and analysis, decision to publish or preparation of the manuscript.

## Notes

**Mark Fiers** is an expert bioinformatician and core facility manager at the VIB Center for Brain and Disease Research with a strong focus on single-cell analyses.

## Notes

**Liesbeth Minnoye** has an MSc degree in Bio-Engineering and is currently a PhD student in the Aerts laboratory working on scATAC-seq in melanoma.

## Notes

**Sara Aibar** is a computer engineer with a PhD in Bioinformatics. As postdoc in the Aerts laboratory, she is the developer of SCENIC, a method to infer GRNs from scRNA-seq data including TF motif discovery.

## Notes

**Carmen Bravo González-Blas** has an MSc degree in Bioinformatics and is currently a PhD student in the Aerts laboratory developing new bioinformatics algorithms to discover regulatory topics from scATAC-seq data.

## Notes

**Zeynep Kalender Atak** is a postdoc in the Aerts laboratory specialized in cancer regulatory genomics.

## Notes

**Stein Aerts** is a professor at the KU Leuven and VIB Group Leader; his research is focused on decoding enhancer logic and understanding how transcriptional programmes drive dynamic changes in cellular states.
